# Parkinson's Disease: Low-Dose Haloperidol Increases Dopamine Receptor Sensitivity and Clinical Response

**DOI:** 10.1155/2014/684973

**Published:** 2014-11-20

**Authors:** Craig J. Hudson, Philip Seeman, Mary V. Seeman

**Affiliations:** ^1^Department of Psychiatry, Alexandra Marine and General Hospital, 120 Napier Street, Goderich, ON, Canada N7A 1W5; ^2^Clera Inc., 260 Heath Street West, Unit 605, Toronto, ON, Canada M5P 3L6

## Abstract

*Background*. It is known that ultra-low doses of haloperidol can cause dopamine supersensitivity of dopamine D2 receptors and related behaviour in animals. *Objective*. The objective was to determine whether a daily ultra-low dose of 40 micrograms of haloperidol could enhance the clinical action of levodopa in Parkinson's disease patients. *Method*. While continuing their daily treatment with levodopa, 16 patients with Parkinson's disease were followed weekly for six weeks. They received an add-on daily dose of 40 micrograms of haloperidol for the first two weeks only. The SPES/SCOPA scale (short scale for assessment of motor impairments and disabilities in Parkinson's disease) was administered before treatment and weekly throughout the trial. *Results*. The results showed a mean decrease in SPES/SCOPA scores after one week of the add-on treatment. *Conclusion*. SCOPA scores decreased after the addition of low-dose haloperidol to the standard daily levodopa dose. This finding is consistent with an increase in sensitivity of dopamine D2 receptors induced by haloperidol. Such treatment for Parkinson's disease may possibly permit the levodopa dose to be reduced and, thus, delay the onset of levodopa side effects.

## 1. Introduction

While levodopa is the mainstay treatment of Parkinson disease, long-term use of levodopa has many side effects [1]. It is desirable, therefore, to find new ways of enhancing the clinical efficacy of levodopa so as to delay its progressive dose increase over the duration of treatment.

A possible method of enhancing the action of dopaminergic therapy is to increase the sensitivity of dopamine D2 receptors that are the main targets for dopamine agonists [2]. For example, very low doses of dopamine antagonists can increase the number of D2 receptors that are in the high-affinity state for dopamine, known as D2High receptors [3]. Such an increase in brain striatal D2High receptors is consistently associated with increased motor activity in animals [4, 5], indicating increased behavioural sensitivity.

For example, Alttoa et al. [4] found that spontaneous exploratory behavior was related to the proportion of D2 receptors in the high-affinity state in laboratory animals. On the basis of their initial spontaneous exploratory behavior, one-month postnatal rats were divided into high- and low-curiosity groups. The low-curiosity group showed a level of high-affinity D2 receptors that averaged 22.5% of the total D2 population of receptors, while the high-curiosity animals revealed a D2High receptor level of 43.8%. These results suggest a relationship between the D2High state and the increased motor and exploratory behavior in rats.

In another study [5], rats were administered haloperidol i.p. at a dose equivalent to 0.4% of the human dose for its approved indications (psychosis). After several successive days, the high-affinity D2 receptors increased by more than 50%, resulting in a level approximately twice that of untreated animals. The animals exhibited heightened locomotion, active exploration, and increased grooming behavior. These results were still demonstrable for several days after dosing was stopped.

These studies support the hypothesis that an increased number of D2 receptors in the high-affinity state correlates with increased motor behavior [3–5]. In fact, during the course of the present clinical study, Dias et al. reported that low-dose haloperidol had a dopaminergic-like action in animals [6].

## 2. Objective of the Present Study

The present study examined whether an ultra-low daily dose of 40 micrograms of haloperidol could enhance the action of the patient's daily dose of levodopa.

In order to establish the appropriate dose of haloperidol, the following estimates were made. For the treatment of psychosis, the recommended dose for oral haloperidol is 2–10 mg daily. It has been shown that subjects receiving 2 milligrams/day of haloperidol achieve plasma levels of about 1 to 1.5 nanograms per mL of plasma [7]. In order to prevent extrapyramidal side effects, we estimated that the dose to be used in this study (40 micrograms per day) would result in a concentration of 0.03 nanograms per mL of plasma [7, 8].

## 3. Methods

### 3.1. Ethics

Before starting the study, the clinical study protocol, informed consent document, and all other relevant documents were submitted to and approved by the IEC/IRB (Internal Ethics Committee, and Internal Review Board) at the Alexandra Marine and General Hospital (AMGH). The documents were also submitted to and approved by the Therapeutic Products Program at Health Canada. The short name of the trial was CLR4001. At the start of the study, each subject was informed of the title of the study, that the study had been approved by the Therapeutic Products Program at Health Canada in Ottawa, the name of the drug (haloperidol) that would be added to their present medication, the dose of the medication, that the low dose of the added drug was far lower than is normally used for other clinical indications and was not expected to cause any muscle stiffness or related motor effects, and that the subject was free to stop taking the medication at any time. Informed signed consent was obtained from all participants. At the end of the study, the individuals were told by the study physician and the assistant whether the subject had any significant alterations in his/her clinical assessment.

### 3.2. Methods

This was a Phase IIa study intended to examine whether 40 micrograms of haloperidol per day would enhance the effectiveness of the regular daily dose of levodopa in treating Parkinson disease patients. The participants' daily doses of levodopa ranged from 75 to 1,200 mg per day (in the forms of Sinemet or Apo-Levocarb) and were fixed during the study. The average levodopa dose for all patients was 502 mg/day. This procedure, therefore, had patients serve as their own controls. After approval by Health Canada and the AMGH review board, patients being treated for Parkinson's disease were recruited by word of mouth from local general practitioners who had made the original diagnosis of Parkinson disease and had it confirmed by a neurologist using the standard UK Brain Bank criteria for Parkinson's disease. The general nature of the study was described to potential participants by the study coordinator, and written consent was sought. Participants continued with their regular levodopa treatment throughout the course of the trial, and the dose was kept constant.

### 3.3. Inclusion/Exclusion Criteria

To be included, participants needed to have a clinical diagnosis of Parkinson's disease. The diagnosis of Parkinson's disease was made by the family doctor initially and confirmed by a consulting neurologist. While it would have been best in retrospect to select patients early in their illness to test the proof-of-principle for this study, it was decided to test all those who were eligible for the study.

### 3.4. Evaluation

Participants were evaluated at baseline and every week throughout the trial using the SPES/SCOPA scale for motor impairment [9].

### 3.5. Treatment

Participants were treated with 40 micrograms of haloperidol per day for two weeks on a daily basis and returned weekly for assessment for a further four weeks. The liquid haloperidol medication was prepared by the hospital pharmacy (40 *μ*g of haloperidol per vial).

The specific steps were as follows.

Week 1: the study purpose and procedure, as above, were explained to the subject and the consent form was signed. A unique subject number was assigned, and the medical history and current medications were recorded. A SPES/SCOPA scale was scored. Each subject received fourteen vials, each of which contained 40 micrograms of haloperidol with instructions to take the contents of one vial at bedtime each evening for a period of two weeks. The subject's number was recorded for each set of vials. Visit 2 was scheduled for 7 to 9 days later.

Week 2: after one week of medication, the SPES/SCOPA scale was scored, the signs, symptoms, and side effects were recorded, compliance was asked about, and the next visit was scheduled. The same was done at weeks 3 to 7, at which time the patients were thanked for their cooperation and received personal feedback.

### 3.6. Statistical Data

Table 1 lists the ages, the genders, the duration of the disease, the medications, the levodopa daily dose, and the baseline value (at week 1) for each of the patients.  Table 2 lists the SCOPA values on each week after the baseline week 1.

The primary goal of the study was to obtain an estimate of the magnitude of the mean difference on the SPES/SCOPA scale between baseline and subsequent visits.

Based on the effect size in the prior animal work, a sample size of 16 subjects was calculated to provide 90% power to detect a difference at the 5% level, using MANOVA.

## 4. Results

### 4.1. Parkinson's Disease Signs and Symptoms

**Figure 1:**
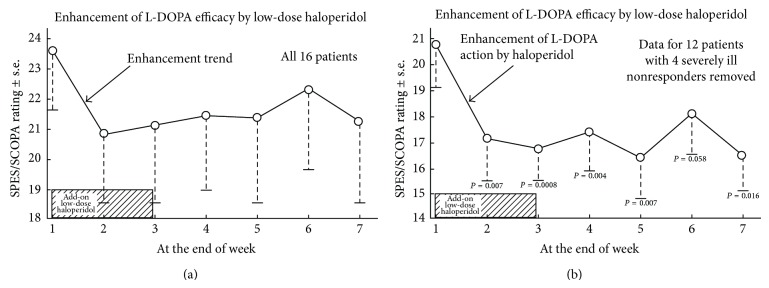
An add-on oral dose of 40 *μ*g haloperidol per day enhanced the effectiveness of levodopa in treating Parkinson disease patients, as monitored by the SCOPA ratings. (a) Data for all 16 patients. (b) Data for 12 patients, where four advanced stage patients were omitted. Data in Tables 1 and 2.

The results of the SPES/SCOPA scale on all 16 subjects in the low-dose haloperidol trial indicated a clear trend of improvement of Parkinson's signs and symptoms, starting after one week of treatment, and continuing to improve over baseline throughout the duration of the trial, as shown in Figure 1(a).

The SCOPA ratings shown in the top of Figure 1 had large values for the standard errors. Although there was a trend toward clinical improvement with haloperidol, the overall effect was modest. Therefore, the vertical scales in Figure 1 were enlarged to show the effects.

### 4.2. Subgroup Analysis

In addition, we speculated whether there might be a sub-group of patients who might have benefitted from the low-dose haloperidol. This was done by omitting four outlier patients who were at the most advanced stage of Parkinson's, as determined by neurological opinions stated in the clinical charts. These four patients (103, 104, 106, and 115) had been taking relatively high doses of levodopa (between 600 and 1200 mg/day, averaging 875 mg/day; the average levodopa dose for the other patients was 366 mg/day). The rationale was that these patients may have had extensive degeneration of their dopamine-sensitive neurons, thus precluding an elevation of D2High receptors secondary to haloperidol. In the remaining subgroup, the average SPES/SCOPA rating at week one (baseline) was 20.8 ± 1.7 (mean ± s.e.), while, after week 2 (after one week of low-dose haloperidol), the average score was 17.1 ± 1.6 (mean ± s.e.), a difference that was statistically significant (*P* = 0.007), based on a one-tailed *t*-test (Table 1, bottom data). The *P* values were obtained by comparing the ratings at each week with the control baseline rating at week 1.

Comparing week 1 and week 3, that is, when the 2-week period of low-dose haloperidol was stopped, the results were even more significant. The average SPES/SCOPA rating at week 1 was 20.8 ± 1.7 (mean ± s.e.), while after week 3 the SPES/SCOPA rating was 16.7 ± 1.2 (mean ± s.e.), with the difference being statistically significant at a *P* value of 0.0008.

As indicated by the mechanism of action of low-dose haloperidol in elevating D2High receptors, the clinical response was highest at the end of the third week. The fall of 4 points on the Parkinson's rating scale suggests a clinical improvement in the patients' signs and symptoms.

### 4.3. Freezing Signs

**Figure 2:**
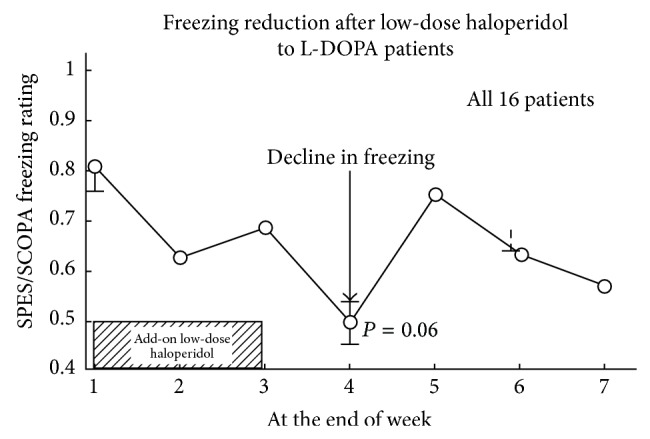
While continuing the daily dose of levodopa, the add-on low dose of 40 *μ*g haloperidol resulted in lower freezing ratings on the SCOPA scale.

The results showed a significant decline of freezing (episodes of inability to move; feet glued to the ground; feet frozen; freezing during speaking, eating, writing, or brushing teeth; freezing while getting out of bed, stepping forward, drinking, or even freezing when thinking) from Week 1 (a rating of 0.81) to Week 4 (one week after stopping treatment)—a rating of 0.50 (Figure 2). The *P* values were obtained by comparing the ratings each week with the baseline rating at week 1. The reduction of freezing upon haloperidol withdrawal was an important unexpected finding.

### 4.4. Safety

The ultra-low dose of 40 *μ*g haloperidol was found to be safe and well tolerated. At each visit, the patients and their companions or spouses were asked whether there were any new symptoms that developed during the week. Other than two participants reporting a subjective worsening of tremors at week 2, there were no instances of any severe worsening of the Parkinsonian signs caused by the added low-dose haloperidol. All reports (e.g., headache, nausea, dizziness, and URI) were tracked and considered unrelated.

## 5. Discussion and Conclusion

The add-on dose of 40 micrograms of haloperidol per day appeared to enhance the clinical action of levodopa, as shown in Figure 1. This effect occurred during the add-on of the low-dose haloperidol, as well as after the haloperidol was stopped.

The synergism between the effect of haloperidol and levodopa is not surprising, because it has long been known that haloperidol can induce dopamine supersensitivity.

However, it should be noted that the haloperidol effect reported here may be within an “inverted U-shaped response.” That is, while very low doses of haloperidol are effective in causing dopamine supersensitivity, higher doses of haloperidol are well known to elicit the opposite effect and exacerbate the Parkinsonian signs.

Many additional patients are needed to extend the present findings and to determine the duration of the add-on effect. It is possible, for example, to give the add-on haloperidol for only one or two weeks each month to enhance the levodopa action.

In addition, a larger study with many more patients would need to examine the test-retest effect after three weeks off haloperidol, in order to see whether the haloperidol potentiation of levodopa action can be reinstated.

Moreover, a larger study will need to examine the period of time for which this haloperidol-add-on approach will continue to generate a positive response.

A longer and more extended study will also need to examine the stage of Parkinson's disease for which this approach is likely to be effective.

Furthermore, the potentiation of the levodopa efficacy by the low-dose haloperidol may depend on the concomitant dose of levodopa. This aspect, too, needs to be examined in a more extended study with more subjects.

While the reduction of freezing (Figure 2) was surprising and unexpected, the reduction occurred a week after withdrawal, consistent with the expected elevation of dopamine D2High receptors by haloperidol [3]. It is important to note that the time course and extent of the elevation of haloperidol-induced elevation of D2High receptors and the total population of D2 receptors are not the same. The D2High receptors are generally elevated in a matter of a few days [3], while the D2 receptors become elevated somewhat later.

Overall, the apparent enhancement of the levodopa action by the low-dose haloperidol could in principle delay the progressive increases in the L-DOPA doses, which are usually found necessary in treating Parkinson diseased patients.

## Figures and Tables

**Table 1:** Patient data.

Subject	Age	M/F	Disease duration, years	SCOPA baseline at week 1	Medication	L-DOPA dose/day
101	70	M	1	14	Sinemet 100/25, 3/day	300 mg
102	59	F	2	25	Levocarb 100/25, 1 qhs prn; Stalevo 150/37.5; 5/day	850 mg
103	81	F	31	42	Sinemet cr 100/25, 5/day; Sinemet 100, 1/day	600 mg
104	80	M	9	26	Levocarb 100/10 2 tabs, 5/day	1000 mg
105	76	M	10	23	Pramipexole 200 mg, 3/day	[=600 mg]
106	70	M	12	29	Apo-Levocarb 100/25, 3 tabs, 4/day	1200 mg
107	81	M	4	20	Apo-Levocarb 25 mg, 3/day	300 mg
108	65	M	10	27	None	0 mg
109	61	M	3	16	Levocarb 25 mg, 4/day	100 mg
110	67	M	8	16	Levocarb 250/25 mg, 4/day; pramipexole 1 mg/day	1000 mg
112	72	M	6	30	Apo-Levocarb 25 mg, 4/day	100 mg
113	61	M	6	21	Sinemet, 100/25, 3/day	300 mg
114	63	M	12	28	Sinemet 100/25, 2 tabs, 5/day	1000 mg
115	77	F	7	30	Sinemet 100/25	700 mg
117	92	M	15	13	Sinemet cr 100/50, 2/day	200 mg
118	80	M	5	17	Apo-Levocarb 100/25, 1/day	100 mg

Average	72		8.8	23.6		
s.e.				1.9		

**Table 2:** SCOPA ratings at the end of each week.

Case	Week 1	Week 2	Week 3	Week 4	Week 5	Week 6	Week 7
101	14	11	10	15	12	17	14
102	25	29	20	25	28	22	19
103	42	46	49	48	52	49	47
104	26	29	32	32	33	34	33
105	23	20	22	22	25	28	23
106	29	25	27	28	30	27	33
107	20	18	17	17	11	15	15
108	27	20	16	17	12	15	10
109	16	14	14	17	16	15	13
110	16	10	15	12	12	14	12
112	30	18	21	24	19	19	16
113	21	16	17	20	19	26	21
114	28	20	21	17	20	20	23
115	30	27	30	28	30	29	28
117	13	9	9	7	7	7	8
118	17	20	18	13	14	18	23

Av.	23.6	18.92	21.1	21.3	21.3	22.2	21.1
S.E.	1.9	2.3	2.4	2.4	2.9	2.5	2.6

Data for subgroup analysis:							
Av.	20.8	17.1	16.7	17.1	16.25	18	16.4
S.E.	1.7	1.6	1.2	1.5	1.8	1.6	1.5
*P*		0.007	0.0008	0.004	0.007	0.058	0.016

(*P* values, using matched, one tail students *t*-test).

Four severe cases (and nonresponders; 104, 106, 103, and 115) were removed for the subgroup analysis.
